# Efficacy of a family systems theory-based integrated care protocol for children with helicobacter pylori-associated gastritis

**DOI:** 10.12669/pjms.42.6.14836

**Published:** 2026-06

**Authors:** Cuihong Zhao, Xiaoyu Song, Tongtong Ma, Dawei Tian, Guoliang Zhang

**Affiliations:** 1 Cuihong Zhao, Department of Pediatrics, Maternity & Child Care Center of Qinhuangdao, Qinhuangdao 066000, Hebei, China; 2 Xiaoyu Song, Department of Pediatrics, Maternity & Child Care Center of Qinhuangdao, Qinhuangdao 066000, Hebei, China; 3 Tongtong Ma, Department of Pediatrics, Maternity & Child Care Center of Qinhuangdao, Qinhuangdao 066000, Hebei, China; 4 Dawei Tian, Department of Pediatrics, Maternity & Child Care Center of Qinhuangdao, Qinhuangdao 066000, Hebei, China; 5 Guoliang Zhang, Department of Pediatrics, Maternity & Child Care Center of Qinhuangdao, Qinhuangdao 066000, Hebei, China

**Keywords:** Family Systems Theory, Gastritis, Helicobacter pylori, Integrated Care Protocol

## Abstract

**Objective:**

To investigate the outcome of a Family Systems Theory-Based integrated care protocol in children with Helicobacter pylori(Hp)-associated gastritis.

**Methodology:**

A retrospective analysis was performed on 180 children with Hp-associated gastritis at Maternity & Child Care Center of Qinhuangdao enrolled between January 2024 to June 2025. Based on the type of care intervention received, the children were divided into two groups via a 1:1 matching protocol. The control group(n=90) received conventional care, whereas the observation group (n=90) received the integrated care protocol based on Family Systems Theory. Compared the medical adherence rate and disease recurrence rate between the two groups of children.

**Results:**

At four weeks after care intervention, the observation group exhibited significantly higher scores for the MUIS-FM dimensions of lack of clarity, unpredictability, information deficiency, and ambiguity compared with the control group(all *P*<0.05). The PedsQL^TM^4.0 scores in the observation group were significantly higher at two weeks and four weeks after care intervention than those in the control group(*P*<0.05). In contrast, the STAI-Y scores in the observation group were significantly lower at two weeks and four weeks after care intervention compared with the control group(*P*<0.05). Furthermore, the observation group had a significantly higher medical adherence rate and a significantly lower disease recurrence rate than the control group(*P*<0.05).

**Conclusion:**

Implementation of the integrated care protocol based on Family Systems Theory for children with Hp-associated gastritis can alleviate the level of illness uncertainty among primary caregivers, improve children’s quality of life, enhance their medical adherence, and reduce the disease recurrence rate.

## INTRODUCTION

Helicobacter pylori (Hp) is a Gram-negative bacillus that primarily colonizes various regions of the duodenum and stomach. Hp-associated gastritis is a common gastrointestinal disease in children.^1^,^2^ Statistics^3^ show that the incidence of this disease has increased year by year. Early-stage disease is characterized by symptoms such as abdominal pain, bloating, nausea, and vomiting, which impair children’s growth, development, and nutritional intake. With disease progression, it may result in serious complications including peptic ulcers and gastric cancer, thereby imposing a substantial physical and psychological burden on both the children and their families. Although triple or quadruple therapy regimens combining proton pump inhibitors, bismuth agents, and antibiotics can effectively eradicate Hp, therapeutic outcomes under conventional care are often suboptimal, and the disease is prone to recurrence due to factors including the young age of the children, inadequate disease-related knowledge, and poor treatment adherence.^4^,^5^

Additionally, Hp is primarily transmitted via the fecal-oral route. Close contact among family members renders the family a key reservoir of infection. Consequently, the family environment plays a critical role in the occurrence, progression, and prognosis of the disease.^6^ Family Systems Theory emphasizes that the family is an interconnected and mutually influential holistic system. Family interaction patterns, emotional support, and behavioral habits all exert a profound influence on an individual’s health. An integrated care protocol developed based on this theory centers on the child within the family system: it delivers comprehensive and continuous care to the child from multiple dimensions by fully mobilizing family resources and encouraging active participation of family members in the care process. While this protocol has demonstrated considerable efficacy,^7^,^8^ limited studies have reported its application in children with Hp-associated gastritis. Therefore, the present study aimed to further investigate the efficacy of the Family Systems Theory-based integrated care protocol in children with Hp-associated gastritis, with the goal of providing evidence for the optimization of future care protocols.

## METHODOLOGY

A retrospective analysis was conducted on the clinical data of 180 children with Hp-associated gastritis who were enrolled between January 2024 to June 2025. Among these patients, 100 were male and 80 were female, with a mean age of (9.56±2.54) years, a mean disease duration of (18.62±3.29) months, and a mean age of primary caregivers of (36.68±3.51) years. Regarding the educational attainment of caregivers: 100 had completed junior college or senior high school, 71 held a bachelor’s degree, and 9 had a master’s degree or higher. The children were stratified into two groups via a 1:1 matching protocol based on the care intervention received(n=90 each group).

### Ethical approval:

The study was approved by the Institutional Ethics Committee of Maternity & Child Care Center of Qinhuangdao (No.: QHDFY-20250600301; Date: June 03, 2025), and written informed consent was obtained from all guardians of the participants.

### Inclusion criteria:

- Fulfilling the diagnostic criteria for Hp-associated gastritis,^9^ presenting with varying degrees of symptoms including epigastric pain, fatigue, anorexia, acid regurgitation, belching, and gastric distension.
- Positive results for the ^13^C or ^14^C urea breath test (UBT).
- Primary caregivers capable of normal communication.
- Complete clinical records of the children.

### Exclusion criteria:

- Comorbid with other gastrointestinal diseases, such as gastric cancer, gastric ulcer, gastroduodenal ulcer, or severe gastric mucosal dysplasia.
- Children with malnutrition.
- Children with precancerous lesions.
- Comorbid with cardiovascular and cerebrovascular diseases.
- Comorbid with other organic disorders
- Primary caregivers with cognitive impairment or abnormalities.

### The control group received conventional care, including:

*Medication guidance:* Prior to discharge, standardized guidelines were distributed to family members, who were instructed on correct medication administration methods, dosages, and timing, as well as potential adverse reactions and corresponding management strategies;

*Lifestyle guidance:* Family members were advised to maintain a regular daily routine for the child, prohibit consumption of cold, greasy, and spicy foods, ensure a balance between rest and activity, and avoid overexertion and mental stress. Additionally, they were instructed to use separate tableware for the child and ensure thorough cleaning of utensils. Meanwhile, close monitoring of disease progression was required, and any abnormalities should be promptly reported to medical staff via telephone or during follow-up appointments.

The observation group received an integrated care protocol based on Family Systems Theory, including the following components:

*Care team establishment*: A multidisciplinary team was formed, comprising two nurses, two dietitians, and one attending physician. All members had at least 3 years of clinical experience and completed standardized training and certification in Hp-associated gastritis management. Nurses were responsible for data collection and follow-up, the attending physician for clinical consultation, and dietitians for developing and guiding individualized dietary plans;

### Intervention implementation:

*a) Health education sessions*: Hp infection-related health education sessions were mandatory for children and their primary caregivers, covering clinical manifestations, risk factors, treatment modalities, and care interventions of Hp infection. A 10-minute interactive Q&A session was conducted post-session, with individualized counseling provided for participants with limited comprehension;

*b) Dietary guidance:* Dietitians developed tailored meal plans based on the child’s habits, dietary preferences, and age. During hospitalization, meals were provided by the hospital nutrition canteen; post-discharge, the plans were executed by primary caregivers;

*c) Emotional intervention:* When interacting with children, caregivers maintained an empathetic and gentle demeanor, encouraging children to express their feelings and facilitating constructive emotional release. Family members received in-depth counseling on the importance of medication adherence to enhance treatment compliance. When feasible, families were advised to engage children in outdoor activities and cultivate hobbies to enrich their daily lives, alleviate anxiety, and foster a supportive home environment;

*d) Regular follow-up:* WeChat-based communication groups were established to disseminate Hp-associated gastritis educational content daily and facilitate peer communication. Telephone follow-up was conducted every 1-2 weeks to monitor the implementation of interventions by family members and address any concerns promptly. The maximum follow-up time for patients in both groups was six months. And case data collection ceased in June 2025.

### Observation indicators:

Both groups received telephone follow-up every one week and on-site or outpatient follow-up every two weeks. Questionnaires were distributed to children and their primary caregivers, with on-site distribution and collection to ensure the validity and completeness of the questionnaires.

Comparison of the Chinese version of MUIS-FM scores^10^ before care intervention and four weeks after care intervention: Evaluating the caregivers’ lack of clarity (7–35 points), unpredictability (3-15 points), information deficiency (4–20 points), and ambiguity (11–55 points). The scale included 4 dimensions and 25 items; higher scores indicated higher uncertainty in caregivers, with a Cronbach’s α coefficient of 0.923;

Comparison of STAI-Y scores^11^ and PedsQL^TM^4.0 scores^12^ of children before care intervention, two weeks and four weeks after care intervention: The STAI-Y scale evaluated children’s anxiety, including trait and state anxiety subscales, with a total of 40 items (1–4 points per item); higher scores indicated higher anxiety, with a Cronbach’s α coefficient of 0.83. The PedsQL^TM^4.0 scale was suitable for children aged 2–18 years, including 23 items divided into four dimensions (role, social, emotional, and physical), with a maximum score of 100 points; higher scores indicated better quality of life, with a Cronbach’s α coefficient of 0.70; Comparison of medication adherence: Assessed based on adherence to scheduled follow-up visits, healthy lifestyle practices, dietary recommendations, and correct medication regimens. The maximum score was 100 points, with ≤60 points indicating non-adherence, 61–80 points indicating partial adherence, and >80 points indicating complete adherence. Overall adherence rate = complete adherence rate + partial adherence rate.

### Statistical analysis:

Statistical analyses were performed using SPSS version 27.0. Categorical variables (e.g., medication adherence, disease recurrence rate) were presented as frequencies and percentages (*n*, %) and analyzed using the chi-square (*χ^2^*) test. Continuous variables (e.g., MUIS-FM, STAI-Y, and PedsQL^TM^4.0 scores) were presented as mean ± standard deviation (*x̄*+*s*) and analyzed using independent samples *t*-tests. The significance level was set at α=0.05.

## RESULTS

No statistically significant differences were observed between the two groups regarding gender distribution, child age, disease duration, primary caregiver age, or caregiver educational attainment (all *P*>0.05). Details are presented in Table-I.

**Table-I:** Comparison of General Data Between the Two Groups.

Group	n	Gender (n, %)		Child Age (Years)	Disease Duration (Months)	Caregiver Age (Years)	Educational Background of Caregivers (n, %)		
		Male	Female				Junior College/ Senior High School	Bachelor’s	Master’s or Above
Observation Group	90	49 (54.44)	41 (45.56)	9.36±2.65	18.98±3.32	36.78±3.37	51 (56.67)	34 (37.78)	5 (5.56)
Control Group	90	51 (56.67)	39 (43.33)	9.75±2.43	18.45±3.17	36.51±3.42	49 (54.44)	37 (41.11)	4 (4.44)
*χ^2^/t*	-	0.090		1.060	1.079	0.527	0.278		
*P*	-	0.764		0.291	0.282	0.599	0.870		

No statistically significant differences were noted in MUIS-FM scores between the two groups prior to care intervention (all *P*>0.05). At four weeks post-intervention, the observation group exhibited significantly higher scores for lack of clarity, unpredictability, information deficiency, and ambiguity compared with the control group (all *P*<0.05). Details are presented in Table-II.

**Table-II:** Comparison of MUIS-FM Scores of Primary Caregivers Between the Two Groups (Scores).

Group	n	Lack of Clarity		Unpredictability		Information Deficiency		Ambiguity	
		Before Care Intervention	four weeks After Care Intervention	Before Care Intervention	four weeks After Care Intervention	Before Care Intervention	four weeks After Care Intervention	Before Care Intervention	four weeks After Care Intervention
Observation Group	90	30.45± 2.68	22.16± 2.23*	11.46± 2.29	7.46± 2.36*	15.66± 2.13	9.86± 2.35*	38.31± 2.65	21.16± 2.86*
Control Group	90	30.73± 2.51	25.79± 2.54*	11.59± 2.54	8.98± 2.41*	15.89± 2.26	10.88± 2.16*	38.59± 2.31	23.55± 2.51*
*t*	-	0.719	10.208	0.370	4.254	0.713	3.038	0.750	5.962
*P*	-	0.473	<0.001	0.712	<0.001	0.477	0.003	0.454	<0.001

***Note:***

* Compared with the same group before care intervention, P<0.05.

Repeated-measures analysis of variance (ANOVA) demonstrated that the main effects of time, group, and group-by-time interaction on STAI-Y scores were all statistically significant (all *P*<0.05). Post-hoc pairwise comparisons using the LSD-t test indicated that the observation group had significantly lower STAI-Y scores than the control group at two and four weeks post-intervention (*P*<0.05). Details are presented in Table-III.

**Table-III:** Comparison of STAI-Y Scores Between the Two Groups During Follow-Up (Scores).

Group	n	Before Care Intervention	two weeks After Care Intervention	four weeks After Care Intervention	F_Time_	F_Interaction_	F_Group_
Observation Group	90	60.35±3.65	45.89±3.12*	37.18±3.55*^#^	9247.869	50.847	40.240
Control Group	90	60.84±3.17	49.87±3.55*	40.85±3.62*^#^			
*t*	-	0.960	7.982	6.927			
*P*	-	0.338	<0.001	<0.001	<0.001	<0.001	<0.001

***Note:***

* Compared with the same group before care intervention, P<0.05;

# Compared with the same group at two weeks after care intervention, P<0.05; the same below.

Repeated-measures ANOVA indicated that the main effects of time, group, and group-by-time interaction on PedsQL^TM^4.0 scores were statistically significant (all *P*<0.05). Post-hoc pairwise comparisons using the LSD-t test demonstrated that the observation group achieved significantly higher PedsQL^TM^4.0 scores than the control group at two and four weeks post-intervention (*P*<0.05). Details are presented in Table-IV. The overall medical adherence rate was significantly higher in the observation group than in the control group (*P*<0.05). Details are presented in Table-V.

**Table-IV:** Comparison of PedsQL^TM^4.0 Scores Between the Two Groups During Follow-Up (Scores).

Group	N	Before Care Intervention	two weeks After Care Intervention	four weeks After Care Intervention	F_Time_	F_Interaction_	F_Group_
Observation Group	90	71.56±3.01	82.63±2.11*	89.85±2.33*^#^	6274.954	128.238	41.995
Control Group	90	71.91±3.22	80.15±2.51*	85.63±2.19*^#^			
*t*	-	0.766	7.224	12.667			
*P*	-	0.445	<0.001	<0.001	<0.001	<0.001	<0.001

**Table-V:** Comparison of Medication Adherence Between the Two Groups (n, %).

Group	n	Complete Adherence	Partial Adherence	Non-Adherence	Medical Adherence Rate
Observation Group	90	49 (54.44)	39 (43.33)	2 (2.22)	88 (97.78)
Control Group	90	38 (42.22)	41 (45.56)	11 (12.22)	79 (87.78)
χ*^2^*	-				6.716
*P*	-				0.010

**Fig.1:**
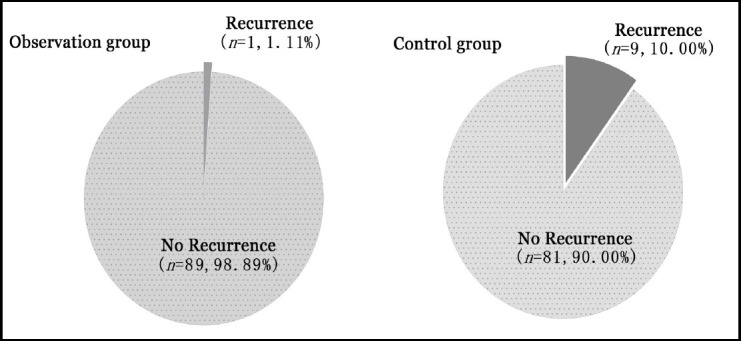
Comparison of recurrence rates between the two groups after six month follow-up.

During the six-months follow-up period, one patient (1.11%) in the observation group experienced disease recurrence, compared with nine patients (10.00%) in the control group. A statistically significant difference in recurrence rate was observed between the two groups (*χ^2^*=6.776, *P*=0.009). Details are presented in Fig.1.

## DISCUSSION

Our results demonstrated that following intervention, the observation group exhibited higher MUIS-FM and PedsQL^TM^4.0 scores, alongside lower STAI-Y scores, compared with the control group. This indicates that the Family Systems Theory-based integrated care protocol provided comprehensive health education and informational support, which enhanced caregivers’ disease-related knowledge and understanding, fostered the holistic physical and psychological development of children, and improved their quality of life.^13^ The establishment of an interdisciplinary care team, coupled with systematic dissemination of Hp-related knowledge^14^-^16^, personalized dietary guidance, emotional support interventions, and regular follow-up, effectively elevated disease awareness among family members and mitigated uncertainty stemming from insufficient information. Furthermore, synchronous engagement and collaborative interventions involving family members interrupted intrafamilial Hp transmission. This multifaceted approach delivered holistic care support to children across physical, psychological, and social domains, promoting comprehensive recovery, reducing recurrence, and improving pediatric patients’ physical and psychological status—ultimately forming a virtuous cycle: “improved family function → enhanced child adherence → optimized disease control → reduced family burden”.^17^ Additionally, the higher medication adherence rate in the observation group is consistent with findings from Zurynski Y et al.^18^, further confirming that the integrated care protocol effectively mobilizes family resources. In contrast, the Family Systems Theory-based integrated care protocol adopts a family-centered framework. Through synchronous screening and treatment of family members, as well as collaborative interventions targeting health behaviors, this protocol can interrupt transmission routes, enhance medication adherence, extend the reach of care intervention, promote recovery in pediatric patients, and reduce disease recurrence.^19^

Specifically, dietary plan development and emotional support interventions directly influenced children’s daily behaviors: dietitian-led guidance on regular eating patterns and nurse-facilitated emotional expression reduced treatment interruptions caused by recurrent symptoms. The regular follow-up mechanism—incorporating WeChat group information sharing and telephone monitoring—established a sustained family support network, promptly addressing treatment-related concerns, strengthening families’ confidence in long-term management, and fostering a “family-healthcare team” collaborative relationship. This not only enhanced caregivers’ sense of responsibility but also, through intra-familial supervision and encouragement mechanisms, promoted strict adherence to medical advice (e.g., medication intake, follow-up appointments), significantly improving treatment compliance. From a long-term efficacy perspective, the lower recurrence rate in the observation group fully validates the sustained intervention advantages of the Family Systems Theory-based protocol. By emphasizing joint family participation in dietary adjustments, medication supervision, and emotional support, primary caregivers and children acquired in-depth knowledge of Hp prevention, treatment, and daily management, effectively enhancing overall family disease management capacity, consolidating therapeutic outcomes, and reducing recurrence rates.

### Limitations:

However, this study has several limitations. First, the sample size was relatively small, which may limit the generalizability of our findings. Second, we did not perform a stratified analysis of intervention efficacy across different family structures or cultural backgrounds. Third, a single center, reliance on questionnaires for data, lack of blinding.

## CONCLUSIONS

The Family Systems Theory-based integrated care protocol for children with Hp-associated gastritis enhances primary caregivers’ disease-related knowledge, improves pediatric patients’ quality of life, alleviates anxiety, increases medication adherence, and reduces long-term recurrence rates.

### Recommendations:

Future research should expand the sample size and explore the applicability of Family Systems Theory across diverse family contexts to further optimize the care protocol.

### Authors’ Contributions:

**CZ:** Literature search, carried out the studies, participated in collecting data, drafted the manuscript, are responsible and accountable for the accuracy and integrity of the work.

**XS** and **TM:** Performed the statistical analysis and participated in its design.

**DT** and **GZ:** Participated in acquisition, analysis, or interpretation of data and drafting of the manuscript.

All authors have read and approved the final manuscript.
